# Implementation of Delayed Cord Clamping in public health facilities: a case study from India

**DOI:** 10.1186/s12884-022-04771-3

**Published:** 2022-06-01

**Authors:** Archana Chowdhury, Sutapa Bandyopadhyay Neogi, Ved Prakash, Nilam Patel, Kunal Pawar, Vinay Kumar Koparde, Anupriya Shukla, Sangeeta Karmakar, Smitha Chekanath Parambath, Sarah Rowe, Homero Martinez

**Affiliations:** 1 Nutrition International, Delhi, India; 2 International Institute of Health Management and Research (IIHMR)-Delhi, Delhi, India; 3 Government of Uttar Pradesh, Lucknow, Uttar Pradesh India; 4 Government of Gujarat, Gandhinagar, Gujarat India; 5 Nutrition International, Gandhinagar, Gujarat India; 6 Nutrition International, Lucknow, Uttar Pradesh India; 7 Nutrition International, India, Delhi, India; 8 Nutrition International, Ottawa, Canada

**Keywords:** Delayed cord clamping, Health systems, Health facilities

## Abstract

**Background:**

Global and country specific recommendations on Delayed umbilical cord clamping (DCC) are available, though guidance on their implementation in program settings is lacking. In India, DCC (clamping not earlier than 1 min after birth) is a component in the package of services delivered as part of the India Newborn Action Plan (INAP) supported by Nutrition International (NI) in two states. The objective of this case study was to document the learnings from implementation of DCC in these two states and to understand the health system factors that affected its operationalization.

**Methods:**

Mixed methods were followed. Using the World Health Organization (WHO) Health Systems building blocks as a framework, 20 Key-Informant Interviews were conducted to explore facilitators and barriers to routine implementation of DCC in public health settings. Existing quantitative program data and secondary data from labour-room registers from eight NI- supported districts were analysed to assess the prevalence of DCC implementation in public health systems settings.

**Results:**

A demonstrated commitment from the government to implement DCC at all delivery points in NI supported districts was observed. Funds were sufficient, trainings were optimal, knowledge of the health workforce was adequate and a recording mechanism was in place. According to record reviews, DCC was more likely to happen in facilities that provide Basic Emergency Obstetric services and among normal deliveries. It was less likely to be followed in babies delivered by Caesarean section (OR 0.03; 95%CI 0.02,0.05), birthweight < 2000 g (OR 0.22; 95%CI 0.12,0.47), multiple pregnancies (OR 0.17, 95%CI 0.05,0.63), birth asphyxia requiring resuscitation (0.37; 95%CI 0.26,0.52), and those delivered during day shift (OR 0.59, 95%CI 0.40, 0.83).

**Conclusions:**

Wide coverage of DCC in public health settings in the two states was observed. Good governance, adequate funding, commitment of health workforce has likely contributed to its success in these contexts. These are critical elements to guide DCC implementation in India and for consideration in other settings.

## Introduction

Delayed umbilical cord clamping (DCC) is an essential component of labour management and immediate newborn care. The World Health Organization (WHO) recommends DCC (not earlier than 1 min) after birth to improve maternal and infant health and nutrition outcomes [1]. However, DCC is slow to be adopted into practice despite a substantial body of evidence on its benefits, including helping to ensure adequate infant iron stores in the first months of life, as well as the existence of global and country recommendations. The feasibility of its implementation in routine care has been reported by a few studies from high-income countries [2–5]. These studies primarily assessed the effectiveness of DCC and reiterated that implementation is feasible in tertiary care units. Adoption of this practice requires multidisciplinary approach [6]. Understanding the obstacles to implementation informs strategies to improve coverage of the practice worldwide. However, there remains limited documentation of program experiences that could inform practical guidance in this area, especially in low-and- middle income countries.

The India Newborn Action Plan (INAP) is India’s commitment to the Global Every Newborn Action Plan (ENAP), launched in 2014 at the 67th World Health Assembly, to advance the Global Strategy for Women’s and Children’s Health [7]. The ENAP envisions the elimination of preventable newborn deaths and stillbirths by 2030. INAP lays out a plan for India to end preventable newborn deaths, accelerate progress, and scale up high-impact, cost-effective interventions. Nutrition International (NI) has been supporting the state governments of Gujarat and Uttar Pradesh (UP) to implement the INAP since 2015 under its Right Start Project (2015-2020), funded by the Government of Canada. These two states were selected for their willingness and readiness to work on implementing the INAP.

NI has focused on improving maternal and newborn care services at health facilities and in the community. This includes the entire package of care under the INAP, with specific emphasis on neonatal resuscitation, timely initiation of breastfeeding (TIBF), prevention of hypothermia, vitamin K supplementation, kangaroo mother care (KMC) and DCC. INAP implementation had just begun when NI initiated this project in late 2015, and sensitization in UP and Gujarat had only started. NI’s main role was to provide technical support and assist the health systems to implement INAP.

NI commissioned this case study to an external, independent evaluator, to confirm these early findings, document the learnings and recommendations from its collaboration with the state governments and understand the health system factors that affected operationalization of DCC in UP and Gujarat.

## Methods

### Context

The key activities conducted as part of the larger study were as under:

- *Preparation for implementation*
  NI held orientation meetings with the Ministry of Health and state governments to introduce the first 1000 days approach within the ambit of INAP [7, 8]. The selection of project districts and the implementation plan were finalized mutually with the government.
- *Capacity building of implementers*
  The target group for capacity building included key implementers such as Accredited Social Health Activist (ASHA) Facilitators, Block Coordinators and Program Managers (BCPMs), Lady Supervisors (LSs), doctors and staff nurses. NI supported the creation of a pool of master trainers with the help of state and district level experts, who further imparted trainings to other target groups. All capacity building workshops were held on Government premises with NI funds. NI also developed context specific training materials on Essential Newborn Care (ENBC) and Home-Based Newborn Care (HBNC) building on the existing guidelines in local languages. The core content of the ENBC module was on facility-based newborn care which included DCC.
- *Supportive Supervision and on-the-job mentoring*
  NI developed three checklists to facilitate Supportive Supervision and on-the-job mentoring: an Observation Checklist, a Facility Assessment Checklist, and a Community Checklist. The Observation Checklist was designed to monitor compliance to key labour room practices of functionaries at the facility. The Facility Assessment Checklist was developed to assess the availability of key maternal and newborn care services. The Community Checklist was intended to observe the community level activities and practices undertaken by field-level functionaries. NI project staff observed deliveries on a quarterly basis for L1 facilities and every month for L2 and L3 facilities.^1^ The observations focused on DCC practices, timely initiation of breast feeding and Vitamin K injections. NI compiled these reports from across the state and shared key findings with the state governments to inform program modification and quality improvement. Real-time course correction and mentoring were also done by the project staff wherever feasible. Apart from mentoring, informal interactions and discussions were held with doctors and nurses to understand their knowledge, attitude and practices with respect to newborn care.
- *Routine monitoring and documentation*
  NI established a routine reporting system at the facility level. Block Coordinators were responsible for data collection from all facilities in their respective blocks. Data were collected from labour room registers based on random checks initially documented on hard copies, but gradually transitioned to electronic data capturing through tablets with pre-loaded checklists. These were shared with the respective district teams daily. Key findings and gaps were formally shared with district level stakeholders during the District Health Society meetings. Similarly, consolidated data from all districts were shared with state level officials in UP and Gujarat. Initially the labour room registers lacked an indicator for recording DCC. NI advocated with the states and in 2019, DCC was included as an indicator in all labour room registers in implementation districts. NI also conducted Rapid Assessment Surveys every six months to review and assess all deliveries reported by each state government.

NI’s support to INAP was carried out in three phases: a preparatory phase (December 2015- March 2016), a demonstration phase (October 2016 – March 2018) and a scale-up phase (April 2018 to March 2020). The demonstration phase targeted 286 facilities across five districts and the scale-up phase targeted a total of 1,515 facilities across 28 districts. Facilities included Level 1 (L1), Level 2 (L2) and Level 3 (L3) facilities in public health settings. A comparison of the baseline and endline surveys from the demonstration phase indicated an improvement in DCC compliance across the five districts.

#### Current study

##### Design and setting

A mixed methods designed case study was used to document the implementation of DCC as part of NI’s support to INAP at all primary and secondary health care facilities conducting deliveries in two states.

### Data collection

A researcher, independent of the program team, reviewed and synthesized multiple data sources, and conducted qualitative and quantitative components of the study to gather an in-depth understanding of the process, outcomes, and learnings from NI’s collaboration with the state governments to strengthen operationalization of DCC. WHO health systems model was the guiding framework to aid data collection and analysis. The data sources to supplement primary data were:

- *Existing program information:* Existing information were extracted from the NI Baseline (2016) and Endline (2018) surveys from the demonstration phase; process documentation (2017); Rapid Assessment Surveys (2018–19); and Checklists for onsite observations. This provided background information on the activities based on which topic guides for in depth qualitative study were developed.
- *Existing documentation from monitoring visits*: Documentation reports from NI’s routine monitoring visits to the health facilities, which included direct observation of deliveries, were used. These reports provided valuable insights into implementation bottlenecks over the course of the project. This provided relevant information to support interaction with hospital staff.
- *Secondary data analysis:* Review of routine labour room data collected from eight health facilities between May and July 2019. L2 and L3 facilities were randomly selected from the 28 districts where NI supported implementation of INAP in the states of UP and Gujarat to estimate the prevalence of DCC. This was primarily utilized to analyze the ‘service delivery’ component of the health systems framework.
- *Primary data collection through observations and Key Informant Interviews*: Facility visits and informal interviews with service providers (doctors and nurses) and program managers were conducted. From amongst those who were present on the day of the visit, key informants were selected purposively for interaction. The interviews continued till the point of data saturation (when no new information was obtained), which was reached with 20 interviews.
- A topic guide was developed to guide the discussions on DCC. Discussions were held either individually or in groups and the information was captured in handwritten notes and electronically (wherever feasible). These notes were transcribed by the researcher and used to assess facilitators and barriers and triangulate the information obtained from the secondary data analysis.

### Ethics

Approval was sought and granted from/by Ethics Committees of Indian Institute of Public Health (IIPH) Delhi, Institutional Review Board of Centre for Media Studies, Delhi and Institutional Ethics Committee of Career Institute of Medical Sciences and Hospital, Lucknow. Routinely collected secondary data were analysed after obtaining due permission from state and district authorities. Every information was kept confidential.

### Analysis

To identify the factors that facilitated and constrained implementation of DCC, the findings from the reports (both quantitative and qualitative) were analysed according to the WHO health systems framework [9]. This framework describes health systems in terms of six components, “building blocks” which include: Leadership and governance, Health system financing, Medical products, vaccines and technologies, Health information systems, Health workforce and service delivery. The interviews focusing on each building block were transcribed verbatim, translated in English, and coded manually. A reflexive and inductive approach was used to code the responses, allowing the codes and categories to emerge from within the data that were later categorized into thematic areas pertaining to health systems building blocks.To support the findings under service delivery, quantitative data from the secondary data analysis were examined to provide descriptive information on the prevalence of DCC in different types of facilities. Association of different factors with prevalence of DCC was determined as bivariate analysis and expressed as Odds Ratios (OR) with 95% confidence intervals. A *p* < 0.05 was selected to establish statistical significance. SPSS version 21.0 was used for the analysis.

## Results

The findings are structured in the context of the WHO health systems building blocks. The findings under the sections of Leadership and Governance, health system financing, health information system and health workforce emerged from the interviews of the key informants. For service delivery component, results from the quantitative assessment and in-depth interviews were used to synthesize the findings.

### Leadership and governance

A high level of commitment for INAP by the central and state Governments was evident. Every district had a nodal person (either the Medical Superintendent or a senior Medical Officer) to oversee INAP activities including DCC. Interviews and discussions with various cadres of officials indicated a sense of accountability for INAP that prevailed within the health system. No development partner other than NI and UNICEF seemed to be engaged in implementation activities in the districts where NI was functioning. While speaking to the administrative staff in the public sector, it was clear they were aware about DCC and the benefits thereof. They were confident that their districts had near universal coverage of DCC (wherever indicated) in public hospitals. This perception aligns well with the information obtained from data from labour rooms (DCC was practiced in 95% of normal deliveries).

All the respondents mentioned that district or state officials routinely visited the health facilities, which indicated a system for monitoring existed in every district. However, there was divided opinion on the focus of DCC during these visits. While some respondents mentioned that supervisors cross-checked their practices on DCC, others denied it. According to them, the discussions mostly revolved around KMC, breast feeding or stillbirths.

Involvement of the private sector seemed to be a grey area in the health system’s implementation of INAP activities. In the catchment areas for L1 and some L2 facilities in remote districts, very few mothers went to private sector hospitals for delivery. However, in areas where L3 facilities are located, several private clinics catered to the delivery load for the district. There was no accountability within the health system for the private sector, as expressed by the respondents during the interviews. There was no mechanism of monitoring or tracking their activities. The Government had devised its own strategies to inform private care providers about DCC through periodic sessions organized by professional organizations. However, district officials mentioned there was no way to verify or document their practices.

In UP, a Medical Officer said, “*Private facilities do not want to share their data. Almost 25% of deliveries happen in private facilities.” (R16_2240919).*

### Health system financing and access to medical products, vaccines, and technologies

All the respondents highlighted the adequacy of Government funds to implement INAP activities. Few trainings in the initial phase were supported by NI. DCC as a stand-alone component requires limited funds apart from capacity building of staff and monitoring. None of the respondents mentioned any shortages for medicine or equipment for essential newborn care such as antenatal corticosteroids, and Vitamin K. All the districts had switched over to an online system of procurement and indenting making it more transparent and accountable. Sufficient stocks were available with the (Community Health Centre) CHCs and Primary Health Centre (PHCs) to meet their demands. This information was also obtained from the periodic rapid assessments.

### Health information systems

There was a well-defined mechanism to collate routine data on INAP at every level and transmit it to upper levels (districts and state). Data from the facilities were compiled at the district headquarters which then forwarded it to the state headquarters. In all the facilities visited, there was a system to record data on DCC in labour room registers. According to the respondents, soon after their trainings (some of which were supported by NI), nurses started documenting the duration of DCC, even though there was no provision in the existing registers to record the time. With the support of NI, at present every labour room register has a designated space to document the duration of DCC.

The development partners supported the health systems in monitoring. As informed by one of the senior officials of a district, *“At the district we track INAP indicators like KMC, SNCU, NBSU, Antenatal corticosteroids, DCC. I have up to date information on all of these indicators. Last month Vit K was given to 87% of the newborns and DCC done on 85% newborns in my district. As part of the Quality Assurance process, we randomly pick few variables and cross check. Monitoring is done by NI. They share their feedback and reports with us. This helps us to identify the gaps and we take measures to address them.” (R9_1200919).*

### Health workforce

All the nurses interviewed mentioned that they were trained on DCC. This component was covered as part of other routine national and state training programs. Those who were not trained formally were trained on-the-job by their peers. Some doctors however highlighted that they could not be relieved from their duties due to other priorities and hence were unable to attend any training workshops. A respondent holding an administrative position opined that the Government organizes the training programs, and they involve partners like NI for delivering some sessions such as DCC. This ensures involvement of partners, improves ownership and support for Government led activities. Absence of refresher trainings was found to be a gap.

The nurses were clear about their roles in the labour rooms with respect to DCC. They seemed to have imbibed DCC as a routine practice. Every labour room had a clock mounted on the wall which helped with recording and documenting the exact time of the umbilical cord clamping.

NI team supported the structured system of supportive supervision by the Government to augment efforts. As expressed by one of the administrative officials, *“I monitor data from the district. NI also does monitoring and gap analysis periodically. They share the report with us which we review. I rely more on NI data as the block coordinators look for specific points as per their agenda”.*

There is, however, no formal mechanism to cross check the records. During the facility visits, we came across few cases, all singleton deliveries, where the timing of cord clamping was 10–12 min. Whether that was an error in recording is still questionable.

### Service delivery

All the staff nurses interviewed were aware about the timing of cord clamping. They all stated 1–3 min as the recommended time or till pulsation stops only if the baby cries immediately after birth. All of them agreed that the timing remains same irrespective of gestational age or birth weight. Only for babies who require resuscitation, DCC cannot be followed.

However, many respondents believed that DCC cannot be done for caesarean sections. This is mainly due to difficulty with finding a proper place to keep the baby. Also, a respondent mentioned that the timing cannot be followed strictly for multiple pregnancies. In some cases, the first baby’s cord is clamped after 15 min.

Direct observation of deliveries in the NI demonstration phase, as per the endline survey showed proportion of deliveries where DCC was performed was 74% (*n* = 92) compared to 41% (*n* = 86) in the baseline (data not shown). Findings from NI’s rapid assessment surveys for the scale-up phase demonstrated an overall increase in DCC practice over a nine-month period (Table 1).

**Table 1:** Proportion of facilities where DCC was followed: findings from NI’s rapid assessment surveys in UP and Gujarat as part of the scale-up phase of the Right Start project

	Oct-Nov 2018		Jun- Jul 2019		Jan-Feb 2020	
Scale up	Number of facilities surveyed	Proportion of deliveries where DCC was followed	Number of facilities surveyed	Proportion of deliveries where DCC was followed	Number of facilities surveyed	Proportion of deliveries where DCC was followed
**UP**						
L1 facilities	648	40.5%	719	43.5%	714	55%
L2 facilities	241	57%	255	70.5%	146	81.5%
L3 facilities	59	58%	63	78.5%	65	90.5%
**Gujarat**						
L1 facilities	69	70%	72	83%	72	92%
L2 facilities	132	81%	128	89%	128	94.5%
L3 facilities	64	71%	68	88%	68	92%

The secondary data review (that are collected routinely from labour rooms as part of the health systems) from eight facilities (*n* = 2141 deliveries) showed that DCC was performed in 95% of cases. In Gujarat it was 98.2% (*n* = 628) while in UP it was 93.6% (*n* = 1513). To explore further, we performed bivariate analyses with factors related to health systems.

Type of facilities (L2 vs. L3) and mode of delivery (normal vs. caesarean) affected the practice of DCC. DCC was more likely to happen in L2 facilities and among normal deliveries. It was less likely to be followed in babies with Caesarean section (OR 0.03; 95% CI 0.02, 0.05), birthweight < 2000 g (OR 0.22; 95% CI 0.12, 0.47), multiple pregnancies (OR 0.17, 95% CI 0.05, 0.63), birth asphyxia requiring resuscitation (0.37; 95% CI 0.26, 0.52), or those delivered during day shift (OR 0.59, 95% CI 0.40, 0.83) (Table 2).

**Table 2:** Bivariate analysis of factors influencing the practice of Delayed Cord Clamping (DCC) in public health settings

Variables	DCC	No DCC	Odds Ratio (OR) (95% CI)
State			
* UP (n* = *1522)*	1425 (93.6%)	97 (6.4%)	3.8 (2.0, 7.2)*
* Gujarat (n* = *628)*	617 (98.2%)	11 (1.8%)	
Type of facility			
* L2 (n* = *542)*	528 (97.4%)	14 (2.6%)	0.44 (0.25, 0.78)*
* L3 (n* = *1606)*	1514 (94.3%)	92 (5.7%)	
Type of delivery			
* Normal (n* = *2051)*	1991 (97.1%)	60 (2.9%)	0.03 (0.02, 0.05)*
* Caesarean section (n* = *99)*	51 (51.5%)	48 (48.5%)	
Low Birth weight			
< *2500 gms (n* = *413)*	391 (94.7%)	22 (5.3%)	0.86 (0.53, 1.38)
> */* = *2500gms (n* = *1721)*	1642 (95.4%)	79 (4.6%)	
Very low birth weight (< 2000 gms)			
< *2000 gms (n* = *78)*	65 (83.3%)	13 (16.7%)	0.22 (0.12, 0.47)*
> */* = *2000gms (n* = *2056)*	1968 (95.7%)	88 (4.3%)	
Preterm			
< *37 weeks (n* = *361)*	339 (93.9%)	22 (6.1%)	1.5 (0.9, 2.5)
> */* = *37 weeks (n* = *1443)*	1383 (95.8%)	60 (4.2%)	
Preterm			
< *34 weeks (n* = *42)*	38 (90.5%)	4 (9.5%)	2.3 (0.8, 6.5)
> */* = *34 weeks (n* = *1762)*	1684 (95.6%)	78 (4.4%)	
Multiple pregnancy			
* Yes (n* = *13)*	10 (76.9%)	3 (23.1%)	0.17 (0.05, 0.63)*
* No (n* = *2129)*	2026 (95.2%)	103 (4.8%)	
Resuscitation required			
* Yes (n* = *58)*	21 (36.2%)	37 (63.8%)	2.67 (1.9, 3.8)*
* No (n* = *2082)*	2012 (96.6%)	70 (3.4%)	
Shift duty			
* Day (8AM- 8PM) (n* = *1185)*	1113 (93.9%)	72 (6.1%)	0.59 (0.40, 0.83)*
* Night (8PM- 8 AM) (n* = *963)*	929 (96.5%)	34 (3.5%)	

^*^p < 0.05

## Discussion

This case study on implementation of DCC through the public health facilities in UP and Gujarat demonstrates strong political commitment and governance at all levels of the health system. The coverage of implementation was 95% as per the review of labour room records. It was less likely to be followed in babies with Caesarean section, birthweight < 2000 g, multiple pregnancies, birth asphyxia requiring resuscitation, or those delivered during day shift, owing to work load or institutional differences between shifts. The approach for collating information was pragmatic in nature that lends itself as a strength to the case study. Most of the information was obtained from existing records and documents. The use of multiple sources supported triangulation of the information. Although this approach decreases the risk of bias that may have occurred with the prospective collection of data as part of this exercise, it is not completely devoid of gaps. Purposive selection of key informants depending on their availability on the day of the interview could have introduced some selection bias. Secondary data have usual challenges of reporting and recording errors and missing values; however, these can be minimized with repeated monitoring and supervision. Information obtained from direct observation of deliveries helped us triangulate the information better. The data are obtained from select facilities of two states. Although the findings may be representative of the states owing to similar practices, these may not be generalizable to other states of the country.

Research from Nepal has highlighted the importance of authorized protocols for successful implementation of any intervention [10, 11]. Despite the availability of evidence on the benefits of DCC, even in well-resourced settings, the implementation in clinical practice was reported as a challenge [12, 13]. In India, inclusion of DCC in national guidelines supported consistent information and coherence within the public health system. This was likely to be a main driver behind achieving a high coverage.

Different Quality Improvement (QI) projects have aimed at improving DCC practices [5, 14, 15]. One such QI model helped a not-for-profit community hospital in the United States of America to increase the coverage from 19.5% in 2013 to 85% in 2017 [14]. This is in contrast to the reported prevalence of 52% among non profit hospitals, 44% among government and 43% among private hospitals in US [16]. In another centre, within a year of implementation, the coverage was reported to be 73% among very LBW babies and 93.7% in all infants less than 37 weeks of gestation [15]. This was achieved by regular engagement, feedback and education with key stakeholders including doctors and nurses. It was felt that a mechanism for routine monitoring was needed to embed DCC in the health system. Notwithstanding the good coverage that DCC has in primary and secondary care health facilities, there are challenges with respect to monitoring and supervision. Supervisory visits do happen but the importance of crosschecking data reported with real time records is yet to be strengthened. There are certain aspects which if validated and implemented can improve the coverage further especially in caesarean sections. For instance, the babies could be kept on the anterior thighs of the mothers as was documented in a recent systematic review [17].

There is some evidence in the literature on the types of health system factors that influence the practice of DCC. Barriers include lack of awareness among staff, professional resistance to change, caesarean section, need for resuscitation, risk of jaundice, polycythemia besides logistical difficulties [6]. Besides, hospital characteristics such as level of facility, number of beds and type of hospital ownership also influence practice. For instance, higher facilities (teaching, non-profit hospitals) were less likely to implement DCC in USA [5, 16]. Similarly, in this case study we found that L-3 facilities equipped to deliver more specialized services had lower coverage of DCC. This could be due to more caesarean sections in these facilities. High risk cases may be a deterrent but there is little evidence to support this theory [10]. The literature suggests that the probability of implementing DCC is higher during night shifts, similar to findings in this case study [10]. The differences could be either because of greater work load due to competing priorities or differences in the institutional systems between shifts. For babies requiring resuscitation, DCC was less likely to be followed. This could be due to more aggressive and prompt measures required for managing a case of birth asphyxia. Caesarean section is a negative factor for predicting DCC as reported [5, 6]. In our case study too, we found similar results. This seems to be more of a feasibility issue rather than technical. Placing the baby on mother’s thighs may overcome this barrier [17]. This, however, should be assessed for inclusion in the current global operational guidelines for standardization. Role of private sector is particularly important when wider coverage is aimed for. Innovative approaches should be devised to increase their involvement in programs.

While it is widely recognized that DCC is more beneficial than Early Cord Clamping (within the first 60 s of birth), the optimal timing of cord clamping continues to be a grey area [1, 17, 18]. A recent study from India recommends delaying clamping until delivery of the placenta [19]. Feasibility of DCC in caesarean deliveries, or in asphyxiated babies needs further exploration. A rational choice between providing immediate neonatal care such as resuscitation with cord intact or after cord clamping should be guided by robust clinical effectiveness studies in low and middle-income countries [17, 20].

### Recommendations

Based on our findings we recommend that programmatic commitment from key stakeholders including the government and implementation research is needed to establish a means to further improve monitoring and supervision in public facilities. Opportunities for incorporating an appropriate indicator in Health Management Information System (HMIS) and explore ways to improve the practice of DCC in caesarean sections should be explored. This case study has documented that DCC is being implemented successfully in NI-supported public health facilities in UP and Gujarat with wide coverage. Good governance, adequate funding and commitment of the health workforce have likely contributed to its success in addition to a focus on monitoring and supervision of DCC within the ambit of INAP. With continued efforts, further documentation of implementation in programmatic settings and generation of more evidence on its feasibility in cases of caesarean sections and birth asphyxia, DCC implementation in health systems could be further strengthened and practical global guidance, applicable to low-and-middle income countries should be generated.

### Key messages:

- Wide coverage of Delayed Cord Clamping was observed in public health system settings in select states in India
- Good governance, adequate funding for the program, and commitment of the health workforce were the main health system factors that influenced the operationalization of DCC
- Further documentation of implementation in programmatic settings and generation of more evidence on feasibility of DCC in cases of caesarean sections and birth asphyxia is required
- Practical global guidance towards DCC implementation applicable to low-and-middle income countries needs to be generated

## Data Availability

The data-sets analysed during the current study are available with Nutrition International. The data-sets analysed during the current study are not publicly available as these are part of a larger project but are available from the corresponding author on reasonable request.

## Abbreviations

ASHA: Accredited Social Health Activist
BCPM: Block Coordinators And Program Managers
CHC: Community Health Centre
DCC: Delayed Umbilical Cord Clamping
ENBC: Essential Newborn Care
HBNC: Home-Based Newborn Care
IIPH: Indian Institute Of Public Health
INAP: India Newborn Action Plan
KMC: Kangaroo Mother Care
LS: Lady Supervisors
NI: Nutrition International
NBSU: Newborn Stabilization Unit
OR: Odds Ratios
PHC: Primary Health Centre
QI: Quality Improvement
SNCU: Special Newborn Care Unit
TIBF: Timely Initiation Of Breastfeeding
UNICEF: United Nations Children’s Fund
UP: Uttar Pradesh
USA: United States Of America
WHO: World Health Organization

## Footnotes

1 L1 facilities manage normal deliveries (e.g. Sub-Centres (SCs). Primary Health Centres (PHCs)). L2 facilities provide Basic Emergency Obstetric services (e.g., 24X7 PHCs and Community Health Centres (CHCs)). L3 facilities provide Comprehensive Emergency Obstetric services (e.g., District Hospitals and Referral Units).
